# Association of Stroke at Young Age With New Cancer in the Years After Stroke Among Patients in the Netherlands

**DOI:** 10.1001/jamanetworkopen.2023.5002

**Published:** 2023-03-28

**Authors:** Jamie I. Verhoeven, Bonnie Fan, Mireille J. M. Broeders, Chantal M. L. Driessen, Ilonca C. H. Vaartjes, Catharina J. M. Klijn, Frank-Erik de Leeuw

**Affiliations:** 1 Department of Neurology, Radboud University Medical Center, Donders Institute for Brain, Cognition and Behavior, Nijmegen, the Netherlands; 2 Department of Neurology, Sint Jans Gasthuis, Weert, the Netherlands; 3 Radboud Institute of Health Sciences, Radboud University Medical Center, Nijmegen, the Netherlands; 4 Dutch Expert Centre for Screening, Nijmegen, the Netherlands; 5 Department of Medical Oncology, Radboud University Medical Center, Nijmegen, the Netherlands; 6 Department of Epidemiology, Julius Center for Health Sciences and Primary Care, University Medical Center Utrecht, Utrecht, the Netherlands

## Abstract

**Importance:**

Stroke may be a first manifestation of an occult cancer or may be an indicator of an increased cancer risk in later life. However, data, especially for younger adults, are limited.

**Objectives:**

To assess the association of stroke with new cancer diagnoses after a first stroke, stratified by stroke subtype, age, and sex, and to compare this association with that in the general population.

**Design, Setting, and Participants:**

This registry- and population-based study included 390 398 patients in the Netherlands aged 15 years or older without a history of cancer and with a first-ever ischemic stroke or intracerebral hemorrhage (ICH) between January 1, 1998, and January 1, 2019. Patients and outcomes were identified through linkage of the Dutch Population Register, the Dutch National Hospital Discharge Register, and National Cause of Death Register. Reference data were gathered from the Dutch Cancer Registry. Statistical analysis was performed from January 6, 2021, to January 2, 2022.

**Exposure:**

First-ever ischemic stroke or ICH. Patients were identified by administrative codes from the *International Classification of Diseases, Ninth Revision*, and the *International Statistical Classification of Diseases and Related Health Problems, Tenth Revision*.

**Main Outcomes and Measures:**

The primary outcome was the cumulative incidence of first-ever cancer after index stroke, stratified by stroke subtype, age, and sex, compared with age-, sex- and calendar year–matched peers from the general population.

**Results:**

The study included 27 616 patients aged 15 to 49 years (median age, 44.5 years [IQR, 39.1-47.6 years]; 13 916 women [50.4%]; 22 622 [81.9%] with ischemic stroke) and 362 782 patients aged 50 years or older (median age, 75.8 years [IQR, 66.9-82.9 years]; 181 847 women [50.1%]; 307 739 [84.8%] with ischemic stroke). The cumulative incidence of new cancer at 10 years was 3.7% (95% CI, 3.4%-4.0%) among patients aged 15 to 49 years and 8.5% (95% CI, 8.4%-8.6%) among patients aged 50 years or older. The cumulative incidence of new cancer after any stroke among patients aged 15 to 49 years was higher among women than men (Gray test statistic, 22.2; *P* < .001), whereas among those aged 50 years or older, the cumulative incidence of new cancer after any stroke was higher among men (Gray test statistic, 943.1; *P* < .001). In the first year after stroke, compared with peers from the general population, patients aged 15 to 49 years were more likely to receive a diagnosis of a new cancer after ischemic stroke (standardized incidence ratio [SIR], 2.6 [95% CI, 2.2-3.1]) and ICH (SIR, 5.4 [95% CI, 3.8-7.3]). For patients aged 50 years or older, the SIR was 1.2 (95% CI, 1.2-1.2) after ischemic stroke and 1.2 (95% CI, 1.1-1.2) after ICH.

**Conclusions and Relevance:**

This study suggests that, compared with the general population, patients aged 15 to 49 years who have had a stroke may have a 3- to 5-fold increased risk of cancer in the first year after stroke, whereas this risk is only slightly elevated for patients aged 50 years or older. Whether this finding has implications for screening remains to be investigated.

## Key Points

**Question:**

What is the association of stroke with a new cancer diagnosis after a first stroke for younger and older patients compared with peers from the general population?

**Findings:**

In this cohort study of 27 616 patients aged 15 to 49 years and 362 782 patients aged 50 years or older, incidence of cancer among the younger group was more than 2 times greater in the first year after ischemic stroke and more than 5 times greater in the first year after intracerebral hemorrhage when compared with the general population. The older group experienced a smaller, but still increased, incidence of cancer.

**Meaning:**

This study suggests that patients aged 15 to 49 years have a 3- to 5-fold increased risk of cancer in the first year after stroke compared with individuals who have not had a stroke.

## Teaser

This cohort study assesses the incidence of new cancer diagnoses after a first stroke, stratified by stroke subtype, age, and sex, compared with incidence of cancer in the general population.

## Introduction

Stroke and cancer are 2 of the most common causes of morbidity and mortality worldwide.^1^ In some cases, the 2 are related, as stroke may be a first manifestation of an underlying occult cancer. Patients with cancer are at increased risk of stroke because of direct tumor effects, prothrombotic effects, and treatment effects.^2,3,4^ Also, many cancers and strokes have joint risk factors, such as smoking and obesity.^4,5,6,7^

A previously unidentified cancer can be identified in 2% to 5% of all patients with ischemic stroke within the first year after stroke, and possibly in even higher percentages of patients after stroke without a clear cause (cryptogenic stroke). This cancer can be considered in hindsight as occult, although already active, at the time of stroke.^3,8^ Cryptogenic stroke is rare among older populations, but it represents up to one-third of all patients aged 18 to 49 years at the time of stroke.^9^ Thus, we hypothesize that the association between stroke and poststroke cancer is most pronounced among younger adults. However, to our knowledge, the literature on cancer among young survivors of ischemic stroke is scarce.^2,10,11^ Moreover, the literature on the incidence of cancer after intracerebral hemorrhage (ICH) is very limited for both younger and older patients.

We therefore assessed age- and sex-specific cumulative incidence rates of first-ever cancer, stratified for organ-specific cancer, in the years after a first-ever ischemic stroke or ICH, compared with the general population.

## Methods

This study was performed according to the guidelines of the the medical ethical review board Arnhem/Nijmegen. No ethical review or individual patient consent was required based on the ethical review requirements of the local research ethics committee and due to the use of deidentified data. All analyses were performed in a secured environment of Statistics Netherlands in accordance with Dutch privacy legislation. This study followed the Strengthening the Reporting of Observational Studies in Epidemiology (STROBE) reporting guideline.

### Participants and Study Design

We created a cohort of patients with first-ever stroke, through linking the Dutch Hospital Discharge Register, the Population Register), and the National Cause of Death Register, all property of Statistics Netherlands and available from 1995 to 2018. The exact linkage procedures have been described previously.^12,13,14^ We included patients admitted between January 1, 1998, and January 1, 2019 (date of last available data), to ensure at least 3 years of available medical history for all patients, to be able to select first-ever and not recurrent events. First-ever stroke was defined as the first known hospitalization with either a primary (main) or secondary discharge diagnosis of ischemic stroke, ICH, or stroke not otherwise specified, based on the *International Classification of Diseases, Ninth Revision* (*ICD-9*) and *International Statistical Classification of Diseases and Related Health Problems, Tenth Revision* (*ICD-10*) codes (eTable 1 in Supplement 1). The *ICD-9* and *ICD-10* codes have been proven reliable for the identification of stroke.^15,16^ The accuracy of *ICD-10* codes has been shown to be 85% accurate for all stroke subtypes,^17^ with 80% of all patients with a stroke not otherwise specified having ischemic strokes (eTable 2 in Supplement 1).^17^ Therefore, ischemic stroke was defined by using the *ICD-9* and *ICD-10* codes for both ischemic stroke and stroke not otherwise specified. To ascertain the expected incidence of age-, sex-, and calendar year–matched peers from the general population, we used the Dutch Cancer Registry. This registry contains all cancer diagnoses from all Dutch citizens between 1989 and 2020 and is 97% complete.^18^

We included only patients aged 15 years or older at first-ever stroke because pediatric stroke differs from stroke in adults in terms of causes and risk factors. We used 15 years as the lower age limit instead of 18 years, which is more common in the literature on stroke among young adults, because reference data from the Dutch Cancer Registry were available only in 5-year intervals.

### Outcomes

Cancer during follow-up was defined as a first-ever hospital diagnosis of any invasive malignant neoplasm after index stroke, excluding primary central nervous system (CNS) cancers, cancers with known CNS metastases at diagnosis, and nonmelanoma skin cancers. Primary CNS cancers and metastases were excluded because of the risk of misclassification of the index stroke. Nonmelanoma skin cancers were excluded because the *ICD-9* and *ICD-10* codes did not enable us to differentiate between squamous cell carcinomas and basal cell carcinomas; basal cell carcinomas are generally excluded from studies investigating cancer incidence.^19^ The cancers were classified as hematologic, lung, breast, gastrointestinal (subdivided into upper gastrointestinal, colorectal, and pancreas or gall bladder), urogenital tract (subdivided into urologic and cancer of male and female genitalia) and “other” (eTable 1 in Supplement 1). When a patient received diagnoses of multiple cancers, only the first diagnosis after stroke was considered.

**Figure 1.**
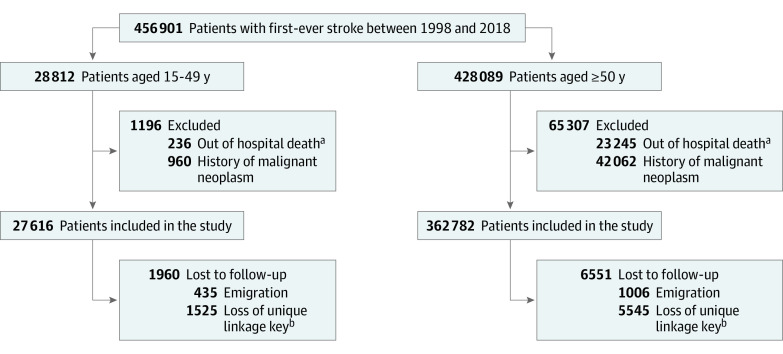
Flowchart of Inclusion and Follow-up ^a^Out of hospital deaths: persons who died from a first-ever stroke without ever reaching the hospital. ^b^Loss of unique linkage key: patients who lost their unique combination of date of birth, sex, and postal code between 1998 and 2013 and could therefore not be reliably followed up over time. After 2013, everyone remained unique due to linkage based on the Dutch Personal Identifier Number.

Patients were followed up from the date of the index stroke to the date of first cancer diagnosis, date of death, date of censoring, or January 1, 2019, whichever came first. Patients were censored if they emigrated during follow-up (1441 of 390 398 [0.4%]), if their personal linkage key became no longer unique and could not be reliably followed over time (7073 of 390 398 [1.8%]) (Figure 1), or if they received a diagnosis of a primary CNS cancer, CNS metastases at time of diagnosis, or nonmelanoma skin cancer.

### Statistical Analysis

Statistical analysis was performed from January 6, 2021, to January 2, 2022. We calculated cumulative incidence with 95% CIs of any cancer, treating death as a competing risk for all patients.^20,21^ We stratified for ischemic stroke and ICH. We also stratified for the following age groups: 15 to 49 years (younger adults) and 50 years or older (older adults), as 49 years of age is a commonly accepted upper age limit in the literature. Subsequently, we divided the younger adults into 2 groups, split close to the median, to differentiate between the rare very young adults with stroke (15-39 years) and the more common 40- to 49-year-old adults with stroke. Finally, we stratified for sex. We tested for differences in cumulative incidences with the Gray test statistic for equality.^22,23^ Person-years of follow-up were calculated for each patient. We also calculated the expected numbers and rates of new cancer per 1000 person-years of any cancer and by cancer subtype based on 5-year age groups, sex, and calendar year–specific cancer incidence rates from the Dutch Cancer Registry, multiplied by the corresponding number of person-years at risk. We calculated standardized incidence ratios (SIRs) and the absolute excess risk of new cancer after stroke by dividing the observed number by the expected number, expressed per 1000 person-years. For calculation of the 95% CIs, we assumed a Poisson distribution.

A 2-sided *P* < .05 was considered statistically significant. For the SIR analyses, we set the threshold for significance to *P* = .006 after Bonferroni adjustment for 8 subgroup analyses. Data analysis was performed using R, version 3.6.2 (Packages: rateratio.test, survival, survminer, cmprsk; R Group for Statistical Computing) and Prism, version 5.03 (GraphPad Software).

## Results

Our cohort consisted of 390 398 patients with a first-ever stroke: 27 616 patients (7.1%) aged 15 to 49 years and 362 782 patients (92.9%) aged 50 years or older (Figure 1). Of the 27 616 younger adults (13 916 women [50.4%]; median age, 44.5 years [IQR, 39.1-47.6 years]), 22 622 (81.9%) had an ischemic stroke and 4994 (18.1%) had an ICH (Table 1). At the end of the follow-up period (median follow-up, 5.7 years [IQR, 2.0-11.7 years]), 3902 of 27 616 patients (14.1%) had died. Of the 362 782 older adults (181 847 women [50.1%]; median age, 75.8 years [IQR, 66.9-82.9 years]), 307 739 (84.8%) had an ischemic stroke, 55 043 (15.2%) had an ICH, and 195 790 patients (54.0%) had died at end of follow-up (median of 2.4 years [IQR, 0.3-5.9 years]).

**Table 1.** Demographic Characteristics of Younger and Older Patients With Stroke, Stratified by Stroke Subtype

Characteristic	Younger adults (15-49 y), No. (%)			Older adults (≥50 y), No. (%)		
	Any stroke (n = 27 616)	Ischemic stroke (n = 22 622)^a^	ICH (n = 4994)	Any stroke (n = 362 782)	Ischemic stroke (n = 307 739)^a^	ICH (n = 55 043)
Sex						
Female	13 916 (50.4)	11 645 (51.5)	2271 (45.5)	181 847 (50.1)	154 265 (50.1)	27 582 (50.1)
Male	13 700 (49.6)	10 977 (48.5)	2723 (54.5)	180 935 (49.9)	153 474 (49.9)	27 461 (49.9)
Age, median (IQR), y	44.5 (39.1-47.6)	44.7 (39.5-47.7)	43.5 (36.3-47.2)	75.8 (66.9-82.9)	75.8 (66.9-82.9)	76.0 (67.0-82.8)
Follow-up						
Median (IQR), y	5.7 (2.0-11.7)	6.0 (2.4-12.0)	3.8 (0.2-10.1)	2.4 (0.3-5.9)	2.7 (0.6-6.2)	0.4 (0.0-4.0)
At ≥5 y	14 746 (53.4)	12 590 (55.7)	2156 (43.2)	107 611 (29.7)	96 333 (31.3)	11 278 (20.5)
At ≥10 y	8627 (31.2)	7371 (32.6)	1256 (25.2)	40 220 (11.1)	35 896 (11.7)	4324 (7.9)
Total No. of person-years	198 215	168 942	29 273	1 413 676	148 425	1 265 251
Cancer during follow-up						
Any cancer	961 (3.5)	837 (3.7)	124 (2.5)	23 371 (6.4)	21 168 (6.9)	2203 (4.0)
Breast^b^	213 (1.5)	188 (1.6)	25 (1.1)	1964 (1.1)	1748 (1.1)	216 (0.8)
Gastrointestinal tract	192 (0.7)	173 (0.8)	19 (0.4)	6660 (1.8)	6123 (2.0)	537 (1.0)
Upper gastrointestinal	45 (0.2)	ND^c^	ND^c^	1498 (0.4)	1374 (0.4)	124 (0.2)
Colorectal	112 (0.4)	ND^c^	ND^c^	4109 (1.1)	3782 (1.2)	327 (0.6)
Pancreas or gall bladder	35 (0.1)	ND^c^	ND^c^	256 (0.1)	196 (0.1)	86 (0.2)
Lung cancer	190 (0.7)	169 (0.7)	21 (0.4)	4393 (1.2)	4012 (1.3)	381 (0.7)
Urogenital tract	139 (0.5)	128 (0.6)	11 (0.2)	5685 (1.6)	5125 (1.7)	560 (1.0)
Urologic tract^d^	68 (0.2)	ND^c^	ND^c^	2673 (0.7)	2423 (0.8)	250 (0.5)
Female genital organs^b^	38 (0.3)	ND^c^	ND^c^	442 (0.2)	399 (0.3)	43 (0.2)
Male genital organs^b^	33 (0.2)	ND^c^	ND^c^	2570 (1.4)	2303 (1.5)	267 (1.0)
Hematologic cancers	92 (0.3)	71 (0.3)	21 (0.4)	1851 (0.5)	1668 (0.5)	183 (0.3)
Other sites^e^	135 (0.5)	108 (0.5)	27 (0.5)	2818 (0.8)	2492 (0.8)	326 (0.6)

Abbreviations: ICH, intracerebral hemorrhage; ND, not disclosed.

^a^ Ischemic stroke was defined by *International Classification of Diseases, Ninth Revision,* codes 434 and 436 and *International Statistical Classification of Diseases and Related Health Problems, Tenth Revision,* codes I63 and I64 for ischemic stroke and stroke not otherwise determined.

^b^ Percentages of breast cancer and cancer of female genital organs are calculated on the total number of women in this group; percentage of cancer in male genital organs are calculated on the total number of men in this group.

^c^ Not disclosed because patient numbers in subgroups were too small to adequately protect privacy according to legislation regarding the use of this register-based data set of Statistics Netherlands.

^d^ Of the 68 urologic cancers in young adults, 34 were located in the kidney and 34 in the bladder or ureter. Of the 2673 urologic cancers in older adults, 718 were located in the kidney and 1955 in the bladder and ureter.

^e^ Cancer of other sites include: melanoma, bone, soft tissue, endocrine organs, head and neck cancers, cancers of miscellaneous sites, cancer with unknown primary location, metastases with an unregistered primary cancer, and multiple primary cancers at the same time.

During follow-up, there were 961 (3.5%) newly diagnosed cancers among the younger group and 23 371 (6.4%) among the older adults (Table 1). Among the younger adults, the 3 most common categories were breast cancer (213 of 961 [22.2%]), gastrointestinal cancer (192 of 961 [20.0%]), and lung cancer (190 of 961 [19.8%]). Among the older patients, the 3 most common categories were gastrointestinal (6660 of 23 371 [28.5%]), urogenital (5685 of 23 371 [24.3%]), and lung cancer (4393 of 23 371 [18.8%]).

### Cumulative Incidence of Any Newly Diagnosed Cancer After Stroke

**Figure 2.**
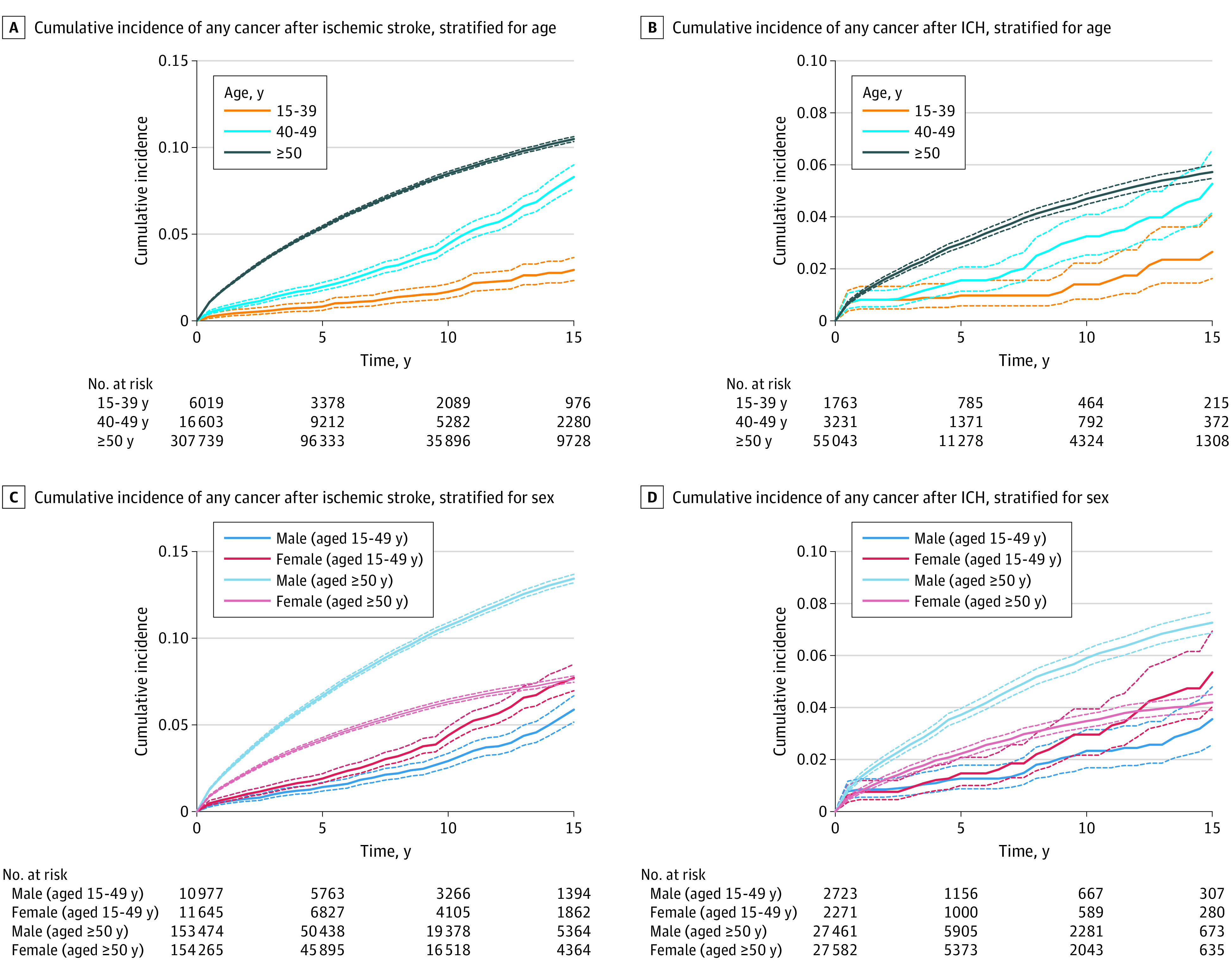
Cumulative Incidence of Any Cancer After Stroke, Stratified by Stroke Subtype, Age, and Sex Dotted lines indicate 95% CIs. ICH indicates intracerebral hemorrhage.

The cumulative incidence of new cancer after any stroke among younger patients was higher among women than men (Gray test statistic, 22.2; *P* < .001). The cumulative incidence of new cancer at 10 years after ischemic stroke was 3.7% (95% CI, 3.4%-4.0%) among younger adults and 8.5% (95% CI, 8.4%-8.6%) among older adults (Figure 2). Among younger patients, women were also more likely than men to receive a diagnosis of a new cancer after ischemic stroke (Gray test statistic, 17.0; *P* < .001). The cumulative incidence of new cancer at 10 years after ICH was 2.6% (95% CI, 2.1%-3.3%) among younger adults and 4.7% (95% CI, 4.5%-4.9%) among older adults. After ICH, there was no difference in the cumulative incidence of new cancer between men and women among the younger group (Gray test statistic, 3.3; *P* = .07).

Among the older group, the cumulative incidence of new cancer after any stroke was higher among men than women (Gray test statistic, 943.1; *P* < .001). Among the older group, men were more likely to receive a diagnosis of a new cancer after both ischemic stroke and ICH (ischemic stroke: Gray test statistic, 1683.6; *P* < .001; ICH: Gray test statistic, 164.8; *P* < .001) (Figure 2).

### Risk of Any Newly Diagnosed Cancer After Stroke Compared With the General Population

**Figure 3.**
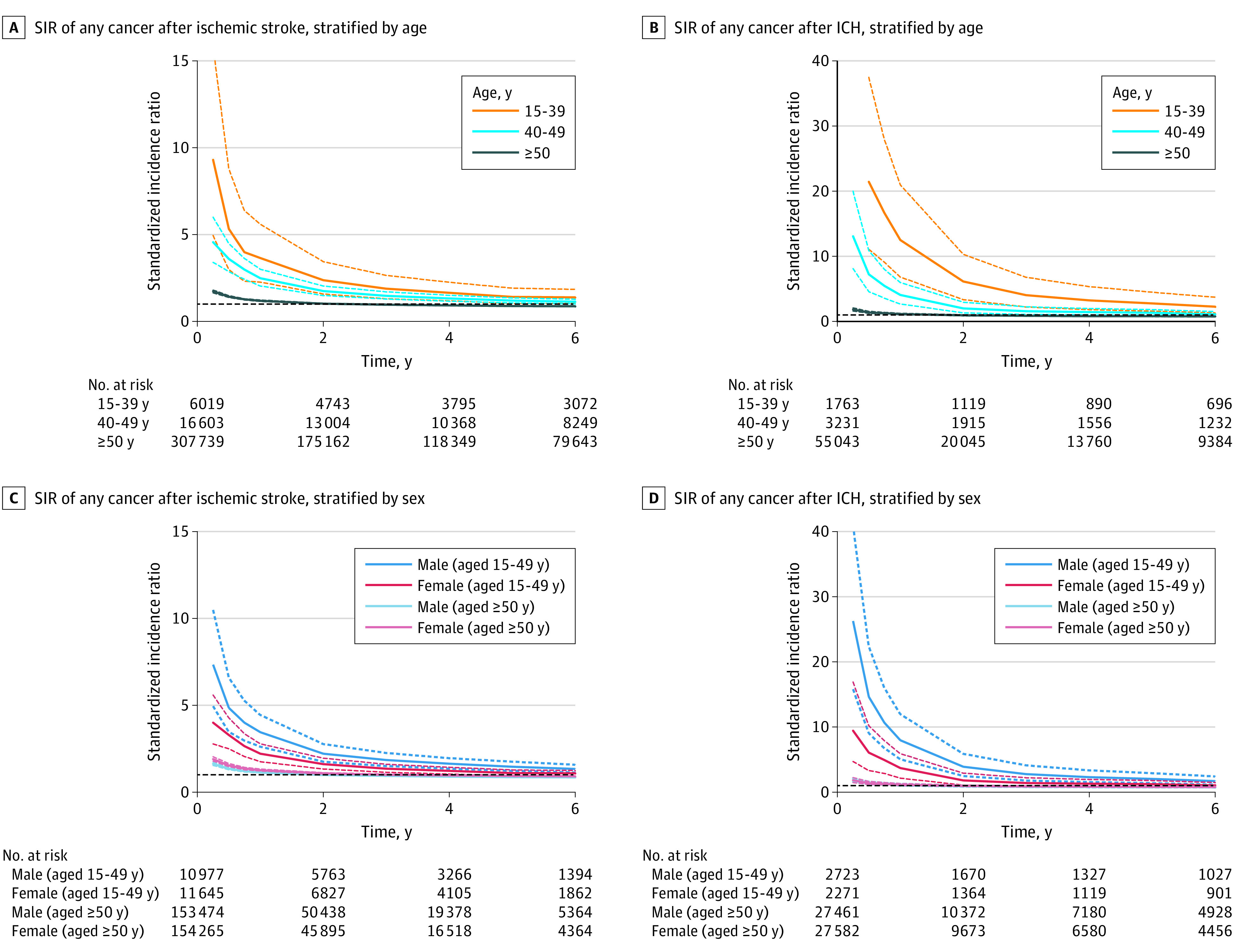
Standardized Incidence Rates (SIRs) of Cancer After Stroke, Stratified by Stroke Subtype, Age, and Sex Dotted lines indicate 95% CIs. ICH indicates intracerebral hemorrhage.

Younger adults were 2.6 (95% CI, 2.2-3.1) times more likely to receive a diagnosis of a new cancer within the first year after ischemic stroke compared with peers from the general population. The cumulative SIR of any cancer decreased over the years but remained significantly increased for 8 years after stroke. After ICH at younger age, the SIR in the first year was 5.4 (95% CI, 3.8-7.3) and remained significantly increased for 6 years after ICH (Table 2). The SIR of new cancer decreased similarly with age after both ischemic stroke and ICH and was only significantly increased for older adults in the first year after stroke. For patients aged 50 years or older, in the first year after stroke, the SIR was 1.2 (95% CI, 1.2-1.2) after ischemic stroke and 1.2 (95%CI, 1.1-1.2) after ICH. The SIRs of new cancer over the years stratified by age and sex are shown in Figure 3.

**Table 2.** SIRs for Developing a New Cancer After Stroke Among Younger Adults (15-49 Years), Stratified by Stroke Subtype, Age, and Sex^a^

Time after stroke	Person-years at risk, No.	Observed events, No.	Observed events per 1000 person-years, No.	Expected events, No.^b^	Expected events per 1000 person-years, No.	Excess event rate per 1000 person-years^c^	SIR (95% CI)^d^	*P* value^e^
**Ischemic stroke^f^**								
New cancer (n = 22 622)								
At 1 y	20 794	134	6.4	51	2.5	3.9	2.6 (2.2-3.1)	<.001
At 5 y	87 465	320	3.7	260	3.0	0.7	1.2 (1.1-1.4)	<.001
Male patients (n = 10 977)								
At 1 y	10 046	58	5.8	16.8	1.7	4.1	3.5 (2.6-4.5)	<.001
At 5 y	41 596	130	3.1	88.2	2.1	1.0	1.5 (1.2-1.8)	<.001
Female patients (n = 11 645)								
At 1 y	10 747	76	3.2	34.2	2.2	3.9	2.2 (1.8-2.8)	<.001
At 5 y	45 869	190	4.1	171.7	3.8	0.4	1.1 (1.0-1.3)	.15
Aged 15-39 y (n = 6019)								
At 1 y	5546	21	3.8	5.7	1.0	2.8	3.7 (2.3-5.6)	<.001
At 5 y	23 740	43	1.8	30.2	1.3	0.5	1.4 (1.0-1.9)	.02
Aged 40-49 y (n = 16 603)								
At 1 y	15 248	113	7.4	45.3	3.0	4.4	2.5 (2.1-3.0)	<.001
At 5 y	63 725	277	4.4	229.7	3.6	0.7	1.2 (1.1-1.4)	.002
Lung cancer (n = 22 622)								
At 1 y	20 794	29	1.4	4.2	0.2	1.2	6.9 (4.7-10.0)	<.001
At 5 y	87 465	68	0.8	24.2	0.3	0.5	2.8 (2.2-3.6)	<.001
Hematologic cancers (n = 22 622)								
At 1 y	20 794	25	1.2	4.8	0.2	1.0	5.2 (3.4-7.7)	<.001
At 5 y	87 465	40	0.5	23.9	0.3	0.2	1.7 (1.2-2.3)	.002
Gastrointestinal tract cancer (n = 22 622)								
At 1 y	20 794	23	1.1	7.3	0.4	0.7	3.2 (2.0-4.7)	<.001
At 5 y	87 465	52	0.6	39.9	0.3	0.3	1.3 (1.0-1.7)	.05
Breast cancer (n = 11 645)								
At 1 y	10 747	17	1.6	19.0	1.8	−0.2	0.9 (0.5-1.4)	.76
At 5 y	45 869	60	1.3	95.9	2.1	−0.8	0.6 (0.5-0.8)	<.001
Urologic tract cancer (n = 22 622)								
At 1 y	20 794	ND^f^	ND^f^	ND^f^	ND^f^	ND^f^	ND^f^	ND^f^
At 5 y	87 465	23	0.3	16.4	0.3	0	1.4 (0.9-2.1)	.09
Female genital tract cancer (n = 11 645)								
At 1 y	10 747	ND^f^	ND^f^	ND^f^	ND^f^	ND^f^	ND^f^	ND^f^
At 5 y	45 869	18	0.4	16.3	0.4	0	1.1 (0.7-1.7)	.57
**Intracerebral hemorrhage**								
New cancer (n = 4994)								
At 1 y	3604	40	11.1	7.5	2.1	9.0	5.4 (3.8-7.3)	<.001
At 5 y	15 158	60	4.0	38.4	2.5	1.5	1.6 (1.2-2.0)	<.001
Male patients (n = 2723)								
At 1 y	2008	23	11.5	2.9	1.4	10.1	8.0 (5.1-12.0)	<.001
At 5 y	8347	31	3.7	15.0	1.8	1.9	2.1 (1.4-2.9)	<.001
Female patients (n = 2271)								
At 1 y	1596	17	10.7	4.6	2.9	7.8	3.7 (2.2-5.9)	<.001
At 5 y	6810	29	4.3	23	3.4	0.9	1.2 (0.8-1.8)	.21
Aged 15-39 y (n = 1763)								
At 1 y	1330	14	10.5	1.1	0.8	9.7	12.5 (6.8-21.0)	<.001
At 5 y	5616	16	2.9	5.8	1.0	1.9	2.8 (1.6-4.5)	<.001
Aged 40-49 y (n = 3231)								
At 1 y	2273	26	11.4	6.4	2.8	8.6	4.1 (2.7-6.0)	<.001
At 5 y	9542	44	4.6	32.6	3.4	1.2	1.4 (1.0-1.8)	.05
Lung cancer (n = 4994)								
At 1 y	3604	ND^f^	ND^f^	ND^f^	ND^f^	ND^f^	ND^f^	ND^f^
At 5 y	15 158	10	0.7	3.6	0.2	0.5	2.8 (1.4-5.2)	.002
Hematologic cancers (n = 4994)								
At 1 y	3604	11	3.1	0.8	0.2	2.9	14.2 (7.1-25.4)	<.001
At 5 y	15 158	13	0.9	3.8	0.3	0.6	3.4 (1.8-5.8)	<.001
Gastrointestinal tract cancer (n = 4994)								
At 1 y	3604	ND^f^	ND^f^	ND^f^	ND^f^	ND^f^	ND^f^	ND^f^
At 5 y	15 158	ND^f^	ND^f^	ND^f^	ND^f^	ND^f^	ND^f^	ND^f^
Breast cancer (n = 4994)								
At 1 y	1596	ND^f^	ND^f^	ND^f^	ND^f^	ND^f^	ND^f^	ND^f^
At 5 y	6810	ND^f^	ND^f^	ND^f^	ND^f^	ND^f^	ND^f^	ND^f^
Urologic tract cancer (n = 4994)								
At 1 y	3604	ND^f^	ND^f^	ND^f^	ND^f^	ND^f^	ND^f^	ND^f^
At 5 y	15 158	ND^f^	ND^f^	ND^f^	ND^f^	ND^f^	ND^f^	ND^f^
Female genital tract cancer (n = 4994)								
At 1 y	1596	ND^f^	ND^f^	ND^f^	ND^f^	ND^f^	ND^f^	ND^f^
At 5 y	6810	ND^f^	ND^f^	ND^f^	ND^f^	ND^f^	ND^f^	ND^f^

Abbreviations: SIR, standardized incidence ratio; ND, not disclosed.

^a^ Absolute numbers and rates are cumulative.

^b^ Expected events retrieved from data of the National Cancer Registry matched for age in 5-year groups, sex, and calendar year.

^c^ The excess event rate was calculated as (observed events − expected events)/person-years at risk, expressed per 1000 person-years.

^d^ The ratio of the observed event rate divided by the expected event rate, assuming the observed events follow a Poisson distribution.

^e^ Two-sided *P* values calculated according to a Poisson distribution. For subgroups of any stroke and subtypes of stroke, the significance threshold was set to a Bonferroni-adjusted *P* < .006.

^f^ Not disclosed because patients numbers in subgroups were too small to adequately protect privacy according to legislation regarding the use of this registry-based data set of Statistics Netherlands.

### Types of Newly Diagnosed Cancer Diagnosed After Stroke Compared With the General Population

For younger adults after ischemic stroke, the risk of newly diagnosed lung cancer (1-year SIR, 6.9 [95% CI, 4.7-10.0]) was highest compared with the general population, followed by hematologic cancers (1-year SIR, 5.2 [95% CI, 3.4-7.7]) (Table 2). The SIRs of lung cancer and of hematologic cancer remained significant for at least 15 and 6 years, respectively, after stroke. Among older adults after ischemic stroke, the SIR of lung cancer (1-year SIR, 1.7 [95% CI, 1.6-1.8]), but not any of the other cancers, was significantly increased compared with the general population. This increased risk remained significant until 15 years after stroke. After ICH, the SIR for younger patients was highest for hematologic cancers (1-year SIR, 14.2 [95% CI, 7.1-25.4]) for at least 15 years after ICH. Among older patients after ICH, the SIR for lung cancer (1-year SIR, 1.6 [95% CI, 1.3-1.9]) was increased for 2 years compared with the general population.

## Discussion

This study, with a large sample size and representative cohort, showed that younger patients with ischemic stroke or ICH were approximately 3 to 5 times more likely to receive a diagnosis of a new cancer in the first year after the stroke compared with peers from the general population. This risk remained increased for 8 years after ischemic stroke and 6 years after ICH. The elevated cancer risk was mainly due to lung cancer, cancer of the gastrointestinal tract, and hematologic cancer. The SIR for newly diagnosed cancer among older patients with stroke was only slightly increased in the first year after stroke.

Previous studies have reported a variable cancer incidence after stroke at all ages: from 1% to 5% at 1 year and between 11% and 30% after 10 years.^19,24,25,26,27,28^ To our knowledge, there have been only 2 studies describing the incidence of poststroke cancer among younger patients. One study described a risk of 0.5% for those aged 18 to 50 years in the first year after stroke,^26^ and another study with a longer follow-up described a cumulative risk of 17.3% in the 10 years after stroke for patients aged 18 to 55 years.^19^ Our findings are at the lower end of this spectrum, which may have several explanations. First, although most of these studies also used a registry-based design,^19,24,26,27^ some used a broader range of *ICD-9* and *ICD-10* codes defining cancer and also included in situ neoplasms.^26,27^ Second, we corrected for death as a competing risk. The short-term and long-term risk of death among the younger patients with stroke,^29^ but even more among the older patients with stroke, is high and may be associated with an overestimation of the cancer risk when not treating death as a competing risk.^23^ Third, we excluded patients with a history of cancer, which eliminated the risk of stroke being the result of previous cancer treatments.^7^

We found that, for younger adults, the SIR remained elevated for more than 5 years after stroke. We found the highest SIR for lung cancer, gastrointestinal cancer, and hematologic cancer. Although the cumulative incidence of breast cancer among young women was the highest, the SIR for breast cancer was not increased and was even significantly decreased at 5 years after stroke. This finding might be explained by the relatively high background risk of breast cancer in the young Dutch population.^30^ The significantly lower risk of breast cancer 5 years after stroke for the younger group may be due to the selection of patients. As breast cancer is relatively common among young women, relatively more women with a history of breast cancer may have been excluded, thereby leaving the women with a possibly lower lifetime risk of breast cancer. It may also indicate that the association between stroke and cancer varies for different types of cancer. This finding highlights the importance of using matched peers as a control group for this type of research.

Although some cancers of the gastrointestinal tract do not equally share smoking as a causative risk factor, most gastrointestinal and lung cancers are strongly associated with smoking, which is a widely accepted risk factor for stroke.^31,32^ This finding may mean that the association of stroke with cancer might be confounded by shared risk factors, such as smoking.^5^ However, this finding cannot explain the increased risk of hematologic cancers because most are not associated with smoking or other cardiovascular risk factors,^6^ nor does it explain why the SIR was much higher for younger compared with older patients with stroke. The difference in SIR between younger and older patients is likely also associated with the higher background incidence of cancer at older ages and the heightened medical surveillance among older people compared with younger people.

Our study shows that the SIR is highest the first year after stroke, with a gradual decrease thereafter, which supports the hypothesis of a causal mechanism between cancer and stroke.^19,25^ It has previously been stated that if cancer is diagnosed within 1 year after stroke, one can assume it was already present at the time of the stroke and may have played a role in causing it.^19^ An important possible causal mechanism is the hypercoagulable state; cancer induces a proinflammatory state that activates the coagulation cascade and increases the risk of stroke.^2,4^ Increased D-dimer and C-reactive protein levels, reflective of a hypercoagulable state, are independently associated with occult cancer among patients with stroke.^33,34^ More specifically, adenocarcinomas produce and release mucin into the bloodstream; mucin is a “sticky” molecule, which triggers the coagulation cascade.^2,4^ Also, there may be direct tumor thrombi due to local invasion of blood vessels or nonbacterial thrombotic endocarditis. Hematologic cancers may cause stroke through, for example, hyperleukocytosis in leukemia and hyperviscosity due to elevated blood protein levels in multiple myeloma.^4^ Finally, a patent foramen ovale may play an important role in enabling a venous to arterial embolism formation because venous embolisms have extensively been associated with active cancer.^2^

We observed a trend toward a higher SIR for cancer after ICH than after ischemic stroke, which was mainly associated with an increased risk of hematologic cancers. To our knowledge, this finding has not been described before, although it has been hypothesized that hematologic cancers may be associated with an increased risk of ICH by affecting coagulation (eg, through causing thrombocytopenia or through hemorrhagic infarcts due to direct tumor thrombi).^2,4^ On the other hand, in some cases bleeding in a cerebral tumor localization may have been misclassified as a primary ICH.

Because the SIR in the younger group was higher compared with the older group, these mechanisms might be more pronounced in younger adults. However, our study design does not allow us to draw conclusions on causal mechanisms. Another explanation for the time association between ischemic stroke or ICH and newly diagnosed cancer may be that patients remained under intensified medical surveillance in the first few years after stroke. However, if this were the only explanation for the increased SIR, we would expect the risk increase to be similar for younger and older patients.

Currently, the diagnostic workup of stroke in young adults includes searching for several rare (clotting) disorders, although screening for cancer is not regularly performed. This study can be considered a stepping stone for future studies investigating the usefulness of screening for cancer after stroke.

### Limitations

Our study has some limitations. First, misclassification may occur when using administrative data. However, previous studies showed good validity of *ICD-9* and *ICD-10* codes for ischemic stroke and ICH.^16,17^ Second, because the Dutch Hospital Discharge Register was available only from 1995 onward, prestroke cancer may have been missed for some patients who had cancer before 1995 and perhaps received therapy that increased stroke risk. Third, we used hospital admissions data to identify a cancer diagnosis during follow-up. Because we had no information on prior outpatient clinic visits, the exact date of diagnosis may vary somewhat from the admission date. Therefore, some of the cancers classified as new after stroke may have already been diagnosed before stroke, leading to some overestimation of early new cancer diagnoses. However, we expect that most (especially younger) patients would have been hospitalized for treatment quickly after receiving their diagnosis. Some cancer diagnoses might have been missed if the the patients were treated without hospital admission, which might have led to an underestimation of cancers diagnosed during follow-up. This scenario applies probably mainly to prostate and bladder cancers and, therefore, the elderly male population.^35^ Also, due to loss to follow-up and patients who were included in the final years of the study with only a short follow-up, we could have slightly underestimated the number of new cancer diagnoses after stroke. Fourth, the lack of clinical data on patient risk factors as well as stroke and cancer characteristics means that we could not further study assumed pathophysiological mechanisms and possible confounding of shared risk factors between (occult) cancer and stroke.

## Conclusions

This cohort study suggests that, especially for younger adults, there is an increased risk of cancer in the first several years, most prominent in the first year, after ischemic stroke and ICH compared with peers from the general population. Large cohorts with sufficient follow-up of extensively phenotyped patients are needed to further clarify the mechanistic link between cancer and stroke, especially among younger adults.

## Notes

Supplement 1. **eTable 1.** Overview of Used *ICD-9* and *ICD-10* Codes **eTable 2.** Results of Validation of Discharge Diagnosis

Supplement 2.
Data Sharing Statement
