# Supplementary material for: Association of Stroke at Young Age With New Cancer in the Years After Stroke Among Patients in the Netherlands
Source: JAMA Netw Open. 2023 Mar 28;6(3):e235002. doi: 10.1001/jamanetworkopen.2023.5002 (PMC10051084; doi:10.1001/jamanetworkopen.2023.5002)
Supplement: Supplement 1. — eTable 1. Overview of Used ICD-9 and ICD-10 Codes eTable 2. Results of Validation of Discharge Diagnosis [file jamanetwopen-e235002-s001.pdf]

Supplementary Online Content

Verhoeven JI, Fan B, Broeders MJM, et al. Association of stroke at young age with new cancer in the years after stroke among patients in the Netherlands. *JAMA Netw Open.* 2023;6(3):e235002. doi:10.1001/jamanetworkopen.2023.5002

eTable 1. Overview of Used *ICD-9* and *ICD-10* Codes
eTable 2. Results of Validation of Discharge Diagnosis

This supplementary material has been provided by the authors to give readers additional information about their work.

© 2023 Verhoeven JI et al. *JAMA Network Open.*

eTable 1. Overview of Used *ICD-9* and *ICD-10* Codes

Diagnosis	*ICD-10*	*ICD-9*
Stroke (exposure)		
Any stroke		
Ischemic stroke	I63, I64	4330 43300 43301 4331 43310 43311 4332 43320 43321 4333 43330 43331 4338 43380 43381 4339 4339 43391 4340 43400 43401 4341 43410 43411 4349 43490 43491 436
Intracerebral hemorrhage	I61	431
Cancer (outcome)		
Hematological cancer		
Hodgkin lymphoma	C81	20140 20141 20142 20143 20144 20145 20146 20147 20148 20150 20151 20152 20153 20154 20155 20156 20157 20158 20160 20161 20162 20163 20164 20165 20166 20167 20168 20170 20171 20172 20173 20174 20175 20176 20177 20178 20100 20101 20111 20121 20102 20112 20122 20103 20113 20123 20104 20114 20124 20105 20115 20125 20106 20116 20126 20107 20117 20127 20108 20118 20128 20110 20120 20190 20191 20192 20193 20194 20195 20196 20197 20198
Non-Hodgkin lymphoma	C82-C88	20200 20201 20202 20203 20204 20205 20206 20207 20208 20040 20041 20042 20043 20044 20045 20046 20047 20048 20000 20001 20051 20071 20002 20052 20072 20003 20053 20073 20004 20054 20074 20005 20055 20075 20006 20056 20076 20007 20057 20077 20008 20058 20078 20050 20070 20010 20011 20012 20013 20014 20015 20016 20017 20018 20020 20021 20022 20023 20024 20025 20026 20027 20028 20030 20050 20080 20031 20051 20081 20032 20052 20082 20033 20053 20083 20034 20054 20084 20035 20055 20085 20036 20056 20086 20037 20057 20087 20038 20058 20088 20210 20211 20212 20213 20214 20215 20216 20217 20218 20210 20220 20221 20222 20223 20224 20225 20226 20227 20228 20220 20270 20271 20272 20273 20274 20275 20276 20277 20278 20270 20060 20061 20062 20063 20064 20065 20066 20067 20068 20283 20280 20281 20282 20283 20284 20285 20286 20287 20288 20380 20382 2733 2038
Plasma cell tumors	C90	20300 20301 20302 20310 20311 20312 20380 20381 20382 2030 2031
Lymphoblastic leukemia/lymphoma	C91	20400 20401 20402 20410 20411 20412 20240 20241 20242 20243 20244 20245 20246 20247 20248 20490 20491 20492 20420 20480 20421 20481 20422 20482 2040 2041 2048 2049
Acute myeloid leukemia	C92	20500 20501 20502 20510 20511 20512 20520 20521 20522 20530 20531 20532 20500 20501 20502 20500 20590 20591 20592 20580 20581 20582 2050 2052 2059

© 2023 Verhoeven JI et al. *JAMA Network Open.*

Myeloproliferative neoplasms	C93-C95	20600 20601 20602 20610 20611 20612 20620 20690 20621 20691 20622 20692 20680 20681 20682 20700 20701 20702 20720 20721 20722 20780 20781 20782 23879 20800 20801 20802 20810 20811 20812 20820 20880 20890 20821 20881 20891 20822 20882 20892 2060 2069 2070 2078 2080 2081 2088 2089
Histiocytic and dendritic cell neoplasms	C96	20250 20251 20252 20253 20254 20255 20256 20257 20258 20260 20261 20262 20263 20264 20265 20266 20267 20268 20290 27789 20291 20292 20293 20294 20295 20296 20297 20298 20230 20231 20232 20233 20234 20235 20236 20237 20238 20291 20292 20293 20294 20295 20296 20297 20298
Breast	**C50**	1740 1750 1741 1742 1743 1744 1745 1746 1748 1749 1759 19881
Gastrointestinal tract		
Upper gastrointestinal tract		
Esophagus	C15	1500 1503 1501 1504 1502 1505 1508 1509
Stomach	C16	1510 1513 1514 1512 1511 1515 1516 1518 1819
Small intestine	C17	1520 1521 1522 1523 1528 1529
Colorectal	C18-C20	1534 1535 1536 1530 1531 1537 1532 1533 1538 1539 1540 1541
Anus	C21	1543 1542 1548
Liver	C22	1550 1551 1552
Gallbladder and bile ducts	C23, C24	1560 1561 1562 1568 1569
Pancreas	C25	1570 1571 1572 1573 1574 1578 1579
Other gastrointestinal	C26	1590 1591 1598 1599 1974 1975 1976 1977 1978
Lower respiratory system		
Trachea, lung and mesothelioma	C33, C34, C45	1620 1622 1623 1624 1625 1628 1629 1970 1971 1972 1973
Urological tract		
Kidney and ureters	C64-C66	1890 1891 1892
Bladder	C67	1880 1881 1882 1883 1884 1885 1886 1887 1888 1889
Other urinary tract	C68	1893 1894 1898 1899 1980 1981
Male genital organs		
Penis	C60	1871 1872 1873 1878 1874
Prostate	C61	185
Testis	C62	1860 1869

© 2023 Verhoeven JI et al. *JAMA Network Open.*

Other male genital organs	C63	1875 1876 1877 1878 1879
Female genital organs		
Vulva & vaginal	C51, C53	1841 1842 1843 1844 1840
Uterus	C53-C55	1800 1801 1808 1809 1821 1820 1828 179
Ovaries	C56	1830 1986
Placental	C58	181
Other female genital organs	C57	1832 1833 1835 1834 1838 1839 1848 1849 19882
Other cancers		
Bone	C40, C41	1704 1705 1707 1708 1700 1701 1702 1703 1706 1709 1985
Kaposi sarcoma	C46	1760 1761 1762 1765 1763 1764 1768 1769
Soft tissues	C38, C47-C49	1641 1642 1643 1649 1630 1631 1638 1639 1648 1649 1718 1580 1588 1589 1710 1712 1713 1714 1715 1716 1717 1718 1719
Thyroid gland	C73	193
Adrenal gland	C74	1940
Other endocrine tumors	C75	1941 1943 1944 1945 1946 1948 1949 1987
Lip	C00	1400 1401 1409 1403 1404 1405 1406 1408
Oral cavity	C01-C06	1410 1411 1412 1413 1414 1415 1416 1418 1419 1430 1431 1438 1439 1440 1441 1448 1449 1452 1453 1454 1455 1450 1451 1456 1458 1459
Salivary glands	C07-C08	1420 1421 1422 1428 1429
Pharynx & larynx	C09-C14, C32	1461 1462 1460 1463 1464 1466 1467 1468 1465 1469 1470 1471 1472 1473 1478 1479 1481 1480 1482 1483 1488 1489 1490 1491 1498 1499 1610 1611 1612 1613 1618 1619
Nasal cavity, middle ear & accessory sinuses	C30, C31	1600 1601 1602 1603 1604 1605 1608 1609
Eye & orbit	C69	1903 1904 1905 1906 1900 1902 1907 1901 1908 1909
Thymus	C37	1640
Other & ill-defined sites	C39, C76	1650 1658 1659 1950 1951 1952 1953 1954 1955 1958 1960 1961 1962 1963 1965 1966 1968 1969 19889
Unknown primary site	C80	1990 1991 1992
Skin		
Melanoma	C43	1720 1721 1722 1723 1724 1725 1726 1727 1728 1729

© 2023 Verhoeven JI et al. *JAMA Network Open.*

Basal cell carcinoma and other skin cancer (excluded)	C44	1730 1731 1732 1733 1734 1735 1736 1737 1738 1739 17300 17301 17302 17309 17310 17311 17312 17319 17320 17321 17322 17329 17330 17331 17332 17339 17340 17341 17342 17349 17350 17351 17352 17359 17360 17361 17362 17369 17370 17371 17372 17379 17380 17381 17382 17389 17390 17391 17392 17399 1982
Central nervous system cancers (excluded)		
Meninges (excluded)	C70	1921 1923
Brain (excluded)	C71	1910 1911 1912 1913 1914 1915 1916 1917 1918 1919
Other central nervous system (excluded)	C72	1922 1920 1928 1929 1983 1984

© 2023 Verhoeven JI et al. *JAMA Network Open.*

eTable 2. Results of Validation of Discharge Diagnosis

Results of validation of discharge diagnosis ICD I64	**N (%)**
total	85
stroke	**74 (87.0)**
ischemic stroke	68 (80.0)
ICH	6 (7.1)
other	**11 (12.9)**
Functional neurological deficit	4 (4.7)
Traumatic ICH	1 (1.2)
TIA	2 (2.4)
Neoplasm	2 (3.5)
CADASIL	1 (1.2)

Legend supplementary table 2:

Abbreviations: N = number, ICH = intracerebral hemorrhage, CADASIL = Cerebral Autosomal Dominant Arteriopathy with Subcortical Infarcts and Leukoencephalopathy.

© 2023 Verhoeven JI et al. *JAMA Network Open.*
