# Supplementary material for: Association of Stroke at Young Age With New Cancer in the Years After Stroke Among Patients in the Netherlands
Source: JAMA Netw Open. 2023 Mar 28;6(3):e235002. doi: 10.1001/jamanetworkopen.2023.5002 (PMC10051084; doi:10.1001/jamanetworkopen.2023.5002)
Supplement: Supplement 2. — Data Sharing Statement [file jamanetwopen-e235002-s002.pdf]

Data Sharing Statement

Verhoeven. Association of Stroke at Young Age With New Cancer in the Years After Stroke Among Patients in the Netherlands. *JAMA Netw Open*. Published March 28, 2023. doi:10.1001/jamanetworkopen.2023.5002

Data
Data available: No

Additional Information
Explanation for why data not available: Individual patient data cannot be shared directly because it is protected data from Statistics Netherlands under legislation of the Dutch Government. Data used in analyses, tables and figures with data dictionary can be made available upon request of a qualified researcher after approval of our institutional review board.
